# Blinding to Circumvent Human Biases: Deliberate Ignorance in Humans, Institutions, and Machines

**DOI:** 10.1177/17456916231188052

**Published:** 2023-09-05

**Authors:** Ralph Hertwig, Stefan M. Herzog, Anastasia Kozyreva

**Affiliations:** Center for Adaptive Rationality, Max Planck Institute for Human Development, Berlin, Germany

**Keywords:** deliberate ignorance, humans, institutions, algorithms, fairness, implicit social biases

## Abstract

Inequalities and injustices are thorny issues in liberal societies, manifesting in forms such as the gender–pay gap; sentencing discrepancies among Black, Hispanic, and White defendants; and unequal medical-resource distribution across ethnicities. One cause of these inequalities is *implicit social bias*—unconsciously formed associations between social groups and attributions such as “nurturing,” “lazy,” or “uneducated.” One strategy to counteract implicit and explicit human biases is delegating crucial decisions, such as how to allocate benefits, resources, or opportunities, to algorithms. Algorithms, however, are not necessarily impartial and objective. Although they can detect and mitigate human biases, they can also perpetuate and even amplify existing inequalities and injustices. We explore how a philosophical thought experiment, Rawls’s “veil of ignorance,” and a psychological phenomenon, deliberate ignorance, can help shield individuals, institutions, and algorithms from biases. We discuss the benefits and drawbacks of methods for shielding human and artificial decision makers from potentially biasing information. We then broaden our discussion beyond the issues of bias and fairness and turn to a research agenda aimed at improving human judgment accuracy with the assistance of algorithms that conceal information that has the potential to undermine performance. Finally, we propose interdisciplinary research questions.

Social-welfare policies can lead to waste, discrimination, resource misallocation, and injustices. One important reason for this is the risk that decision-making could be compromised by implicit biases that infuse public-policy decisions. Implicit biases can surface in attitudes and stereotypes that, possibly without individuals’ awareness, influence social judgments and behavior (Greenwald & Lai, 2020). The concept of implicit bias has gained attention in a range of fields, including psychology, business, law, medicine, and political science, and has sparked initiatives aimed at mitigating its impact (Greenwald et al., 2022). Implicit biases pertain to social categories like gender, ethnicity, sexual orientation, and religion, and can impact decisions that affect people’s welfare and well-being. Many injustices, inequities, and discriminatory behaviors—such as racial bias in police shootings, the gender-pay gap, health-care inequities, and biased hiring practices—are thought to have roots, at least in part, in implicit biases (see Kurdi & Dunham, 2022). Our goal is to explore the potential of blinding individuals, institutions, and algorithms to contain biases’ detrimental impact. We then broaden our discussion beyond the issues of bias and fairness and turn to a research agenda aimed at enhancing human judgment accuracy with the assistance of algorithms that conceal information that has the potential to undermine performance. We first turn to algorithms and their increasingly important role as new decision-making authorities.

## Algorithms as Ubiquitous Decision Makers

Increasingly, collective outcomes are being determined by algorithmic decision-making. Governments around the world use decision-making algorithms for important public-policy decisions (see, e.g., Levy et al., 2021) in domains including law (e.g., predictive policing, recidivism risk, welfare fraud, child-abuse risk), permits (e.g., visa approvals), education (e.g., grade allocation, school admissions), health care (e.g., eligibility for intensive medical care), and social welfare (e.g., benefit-program qualification). Fairness in algorithmic decision-making is vital not only for distributive justice, but also for protecting fundamental rights—particularly online, where algorithmic tools are widely used for content moderation and personalization (see Fig. 4 in Kozyreva et al., 2020; see also Lewandowsky et al., 2020; Lorenz-Spreen et al., 2020). Algorithmic moderation poses risks to human rights, including data privacy (Kozyreva et al., 2021; Wachter & Mittelstadt, 2019) and freedom of expression (Kozyreva et al., 2023). Because algorithms influence decisions with collective implications, it is crucial to ensure that they safeguard human rights and foster fairness and equity. But how?

Generally speaking, an algorithm is a finite sequence of well-defined instructions for processing information given as input (e.g., the attributes of a defendant) to obtain an output (e.g., an estimate of their risk of recidivism). In this article, we primarily discuss algorithms in the form of computer programs, the operation of which may or may not be transparent to humans (Rudin, 2019). However, algorithms can also be instantiated outside of computer programs—for instance, in the human mind or as legal code.

The development of computer algorithms spans a broad spectrum of approaches, encompassing various degrees of human involvement and autonomous machine learning. At one end of the spectrum is a top-down approach in which humans explicitly program the input–output mappings. On the other end is a bottom-up approach known as *machine learning*. The key idea behind machine learning is to allow the algorithm to learn on its own on the basis of a set of example decisions. For instance, to create an algorithm that determines whether a person is eligible for a disability-benefit payment, one could compile a data set of past decisions and derive the algorithm from this historical data. Algorithmic decision-making promises notable benefits: Unlike humans, algorithms are not burdened by fatigue or boredom, and in principle they can process and take into account many more factors in their decisions than the human mind ever could (Swets et al., 2000).

Algorithmic decision-making, however, also carries important risks, including these: First, algorithms can amplify social biases, leading to unfair decisions and perpetuating discrimination and inequality (van Giffen et al., 2022; Wachter et al., 2021). Second, they can manipulate individuals and distort economic and political competitions (Lewandowsky et al., 2020; Lorenz-Spreen et al., 2020). Online platforms’ algorithms, for example, enable advertisers to microtarget users on the basis of personal attributes and vulnerabilities (Lorenz-Spreen et al., 2021). Third, many automated decision-making systems are “black boxes”—complex and hard to understand even for machine-learning experts, making their decisions difficult to trust (Rudin, 2019). Efforts in computer science have focused on making black boxes more interpretable (Dwivedi et al., 2023; Molnar, 2022; Rudin et al., 2022; Speith, 2022), but this is a challenging and controversial endeavor (Miller, 2019; Rudin, 2019). Fourth, the often obscure processes used by algorithms to collect and process personal data pose significant privacy risks (Wachter & Mittelstadt, 2019).

The amplification of bias is an imminent danger in algorithmic decision-making. Biases can enter algorithms through developers’ implicit or explicit biases, or through biased historical training data (for a discussion and classification of biases, see Mayson, 2019; Mehrabi et al., 2021; van Giffen et al., 2022). Empirical evidence suggests that algorithmic decision-making is indeed often biased. For example, Kaushal et al. (2020) analyzed over 70 publications on deep-learning algorithms for image-based diagnostic tasks and found that most algorithms were trained on data from just three U.S. coastal states that vastly differ economically, educationally, socially, and culturally from the remaining 47 states. Therefore, the training data may generalize poorly to other patient populations (Kaushal et al., 2020). Online advertisements also seem prone to biases (Ali et al., 2019; Datta et al., 2018). For instance, Datta et al. (2015) discovered that women were shown fewer Google job ads for highly paid positions compared with men, putting women at a disadvantage for securing high-paying executive jobs.

Unfortunately, for several reasons there are no simple solutions to the problem of biases in algorithms. First, algorithmic bias can take many forms (e.g., measurement bias, label bias, or deployment bias; see van Giffen et al., 2022), making it unlikely that there is only one source of bias at play in any particular setting. Second, the heart of the issue often involves societal biases rather than fixable technical biases in algorithms (Wachter et al., 2021). Third, no single benchmark exists for evaluating algorithmic fairness, and frameworks may conflict or misalign with legal systems (Chouldechova, 2017; Heidari et al., 2018; Kleinberg et al., 2016; Lee et al., 2021; Mitchell et al., 2021; Wachter et al., 2021). Last, concerns other than fairness, such as social welfare and citizen protection, may require difficult trade-offs between fairness, welfare, and autonomy (Lee et al., 2021).

Sobered by these complex difficulties, we now explore a potential path to solutions. For this, we draw inspiration first from the philosopher Rawls’s (1971) idea of how a “veil of ignorance” can help to design a just society, and second from research on the psychological phenomenon of deliberate ignorance (Hertwig & Engel, 2016, 2021).

## The Veil of Ignorance and a Just Society

The justness of a society hinges on how it allocates valued resources such as income, wealth, rights, duties, opportunities, and honors (Sandel, 2009). Thus, the crux of realizing a just society lies in identifying the principles guiding this allocation of resources. Rawls’s theory of justice as fairness (Rawls, 1971; see also Freeman, 2019) proposes that justice emanates from principles agreed upon by individuals placed behind a “veil of ignorance.” In this thought experiment, all parties are placed in a state of ignorance about their positions in society—they are unaware of their and others’ social status, race, gender, assets, abilities, education, and psychological dispositions. They know only the shared characteristics and interests relevant to their roles as free and equal moral persons. Rawls contends that, without any knowledge of where they stand in a society, every rational and moral individual would seek fair principles suitable for a democratic society.

Several objections have been raised against this theory (Freeman, 2019). For one thing, the veil of ignorance is a purely hypothetical construct—in reality, decision makers can never be in this abstract position. Nevertheless, the veil of ignorance offers a valuable framework for establishing fairness principles that benefit most people, irrespective of their individual traits and advantages. Building on this potential, could this concept help to incorporate principles of fairness such as impartiality, equality, and fundamental-rights protection into real-world human and algorithmic decision-making? Before we discuss this possibility for algorithmic decision-making, let us turn to research on deliberate ignorance in humans. In some of its manifestations, the choice not to know embodies some of the function of blinding achieved by Rawls’s veil of ignorance.

## When Deliberate Ignorance Safeguards Fairness

Although human beings have often been portrayed as equipped with a boundless thirst for knowledge (see Hertwig & Engel, 2016, 2021), there are things people prefer not to know. Deliberate ignorance—or the choice to not seek out available information—has recently attracted attention in psychology (e.g., Gigerenzer & Garcia-Retamero, 2017; Hertwig & Engel, 2016), economics (termed “information avoidance”; e.g., Golman et al., 2017), neuroscience (e.g., Charpentier et al., 2018), social sciences (e.g., Gross & McGoey, 2015), and law and public policy (Hertwig & Engel, 2021). Deliberate ignorance is practiced in a range of domains, from medical or genetic information (the “right not to know”; see references and discussion in Berkman & Hull, 2014) to consumer information (e.g., the costs of meat consumption; Kadel et al., 2023). But let us consider deliberate ignorance in a specific context: the social transformation that took place in Germany after the fall of the Berlin Wall. In times of transition and in the face of past misdeeds, issues of fairness, justice, and biases inevitably arise.

The German Democratic Republic (GDR) went to extraordinary lengths to monitor the lives of its citizens and foreign visitors. In addition to full-time employees, vast networks of civilian informants were recruited to spy on colleagues, friends, and even family members suspected of disloyalty. After German reunification, people were allowed to access their files; over 2 million citizens applied to view them. However, it is likely that the majority of those for whom files had been compiled rejected this opportunity. Through surveys and interviews, Hertwig and Ellerbrock (2022) identified a range of reasons behind GDR citizens’ choice to not view their file. One closely related to concerns of fairness and bias. A group of people, including Nobel Prize laureate Günter Grass, wished to protect themselves from knowledge that inevitably would have biased them against the spy in their midst—possibly unfairly, given that it was impossible to know why someone might have informed on them (e.g., was the person coerced, bribed, or ideologically driven?—see also Ellerbrock & Hertwig, 2021).

It is not only individuals who blind themselves to information that may introduce bias—collectives and institutions do so as well (Teichman et al., 2021). For instance, by asking musicians to audition behind a screen so that they cannot be seen, orchestras have increased the proportion of female musicians, mitigating gender-biased practices and boosting female representation (Goldin & Rouse, 2000). Likewise, legal institutions may safeguard impartiality by withholding particular information. For example, in U.S. law, a defendant’s criminal record is considered character evidence, which is inadmissible in criminal proceedings determining guilt (with notable exceptions), even though the criminal record plays a crucial role in determining a convicted defendant’s sentence (Federal Rules of Evidence, Rule 404).

Deliberate ignorance will, of course, not categorically foster fairness, impartiality, or justice. At times, turning a blind eye to information will perpetuate inequality and injustice. For example, child-abuse cases in sports and educative institutions suggest that many in authority willfully ignored troubling information. However, numerous other decisions result in outcomes that are more just and that enhance welfare when the information-processing system is not subjected to the potentially biasing information (Teichman et al., 2021). For this reason, scientific journals implement double-blind review, scientists anonymize scientific data, and discrimination laws forbid requiring photos on job applications (MacCoun, 2021; Robertson & Kesselheim, 2016). Could blinding also serve as a potential solution to algorithmic bias?

## The Difficulties of Circumventing Human Biases by Blinding Algorithms

It seems self-evident that algorithms have the capacity to tackle both implicit and explicit biases because, unlike humans, algorithms can be simply programmed to disregard traits, attributes, and cues that may introduce bias (e.g., name, gender, photos, ethnicity, age). Blinding algorithms to protected attributes should lead them to practice what is essentially a machine version of deliberate ignorance (Teichman et al., 2021). By spreading the veil of ignorance over an algorithm, the hope is that its decision-making will be less biased. Indeed, blinding machine-learning algorithms to specific input information can produce less biased outputs. For instance, Dayanik and Padó (2020) showed how an algorithm processing large amounts of text tends to be better at recognizing statements from individuals who appear more often in the text data that the algorithm was trained on. To fix this, Dayanik and Padó proposed to mask names and pronouns during the program’s training, which helped make the program more fair without affecting its overall performance. As another example, eliminating gendered language from clinical notes mitigates gender bias in medical-classification models without sacrificing accuracy (Minot et al., 2021).

However, blinding algorithms to protected attributes (e.g., race, gender) is not straightforward. For example, a widely used algorithm identifying high-risk patients for resource-intensive care programs exhibited significant racial bias, even though it was blinded to race (Obermeyer et al., 2019). The algorithm relied on health-care costs, rather than illness, as a proxy for health. But unequal access to health care had resulted in less money being spent caring for Black patients than White patients. Thus, using past health-care costs as a health proxy meant that the algorithm reproduced racial bias and penalized Black patients by assigning them lower risk scores than White patients with comparable conditions.

Whenever a protected category such as race can be inferred through proxies correlated with the category, there is a risk of algorithmic bias (Adler et al., 2018; Feldman et al., 2015; Marx et al., 2019; Yeom et al., 2018). This means that explicitly denying access to the protected attribute does not guarantee that it will not influence the decision. For instance, race, a protected attribute, may be linked to postal codes (e.g., because of historical patterns of segregation). If race—for instance, because of bias in policing and the criminal-justice system—is indeed correlated with a particular target variable (e.g., the number of recorded criminal offenses), an algorithm could learn to indirectly infer race by combining one or more proxies (e.g., postal codes) associated with it. The algorithm may thus reproduce bias despite not having direct access to the protected attribute.

The proficiency in spotting shortcuts—a skill shared by humans, animals, and machines alike—can exacerbate the issue of proxy cues. Predictive cues that have only a superficial relationship with the target variable are often exploited (Geirhos et al., 2020). For instance, Zech et al. (2018) examined the performance of convolutional neural networks (CNNs) predicting pneumonia in chest radiographs from patients at three U.S. sites. CNNs trained on parts of a pooled data set displayed superior performance on unseen patients from the same pooled data set relative to CNNs trained on a single site and predicting pneumonia in patients from another site. The reason for this is that the CNNs learned to detect a metal token placed on patients by radiology technicians in a site-specific way. When there is a correlation between an irrelevant feature and the prevalence of disease per site, models can use this confounding information to predict the disease (Zech et al., 2018). This means that the performance of the algorithms in diagnosing diseases on X-rays may reflect not only their ability to identify diseases but also their ability to exploit task-irrelevant information.

Ironically, blinding an algorithm to protected attributes can lead to worse, not better, outcomes for the group needing protection. In some cases, explicitly incorporating the protected attribute can actually lead to outcomes that are more just (Corbett-Davies & Goel, 2018; Simons et al., 2021)—an interesting observation in light of the debate on affirmative action versus “neutral” remedies to discrimination (Dwork et al., 2012). Discussing the case of pretrial recidivism predictions, Corbett-Davies and Goel (2018) observed that women have lower recidivism rates than men even when one controls for typical risk factors such as criminal history, age, and substance use. Gender-neutral risk assessments therefore tend to overestimate the recidivism risk of women. By explicitly incorporating gender as a predictor in the decision-making process, fewer people, particularly women, would be detained—at no cost to public safety.

These two issues—algorithms finding proxies for protected attributes and unintended consequences of not considering protected attributes—highlight that the utmost care is required when blinding machine-learning algorithms to protected attributes. It is crucial to scrutinize the output of both blinded and unblinded algorithms for any biases or harmful downstream effects. A blind algorithm cannot be presumed to be bias-free; it must be empirically demonstrated as such in its natural habitat (Rahwan et al., 2019)—that is, in the situations within which it is designed to operate.

## The Promise of Blinded Algorithms

Merely removing biasing information and then letting algorithms learn is not enough to prevent bias and ensure fairness. Rather, human expertise and values must be explicitly incorporated throughout the entire design process (Birhane et al., 2022) so that problems can be contained from the outset. We will now explore two strategies aimed at achieving this goal. The first approach prioritizes the design of algorithmic systems with a strong emphasis on transparency and simplicity. This not only encourages human comprehension of algorithms, but it also eases the process of algorithmic auditing by experts. The second approach relies on empirical studies involving both general and expert populations to determine the information that should be included or excluded in algorithmic decision processes.

The first promising strategy is to use algorithms that are designed with an emphasis on simplicity and transparency (e.g., simple decision trees or simple tallying models; Hafenbrädl et al., 2016; Katsikopoulos et al., 2021; Keller et al., 2020; Rudin et al., 2022) rather than complex and opaque ones (e.g., random forests or neural networks). Transparent algorithms have the dual advantage of being easier to understand for all kinds of users and easier to audit for bias. The latter point is crucial because even a transparent algorithm may lead to unintended consequences—especially in complex environments. It is therefore always important to audit how systems behave in their operational environment (Rahwan et al., 2019). Furthermore, by carefully deciding which attributes of a case are to be considered or ignored from the outset (i.e., a very deliberate form of “feature engineering”; see Keller et al., 2020), there should be less room for unwanted surprises and it should be easier to audit the algorithm. Simple, interpretable algorithms can take many forms. They can be designed entirely by human experts, without any involvement of machine-learning techniques (e.g., the START decision tree, a triage method used by first responders to quickly classify victims in a mass-casualty incident; Super, 1984). Alternatively, analysts can use machine learning to automatically create algorithms (Rudin et al., 2022)—using a carefully designed set of attributes—and then let human experts select the most suitable one (e.g., from a set of simple decision trees that perform similarly; Wang et al., 2022).

The second way in which human expertise and values could be explicitly incorporated into the design process of algorithms is by empirically studying what information people deem important to ignore and then use these findings to inform how to blind algorithms. This approach can be particularly useful for new and complex moral problems, where no clear ethical benchmarks yet exist and where studying public attitudes ensures that people’s preferences are taken into account—another aspect of fairness.

For example, consider online content moderation, a highly relevant issue in the context of fundamental rights protection. Algorithmic content moderation identifies and sanctions content that is either illegal or in violation of a platform’s policies. Major platforms mostly automate the enforcement of their policies. For instance, a significant proportion of bullying and harassment on Facebook and Instagram is proactively detected by automated tools (Bickert, 2022). Although algorithms execute these decisions, humans design the underlying rules and navigate ambiguities, striking balances between potentially conflicting values. Whether purely algorithmic or involving human oversight, content moderation requires a systematic reconciliation of free-speech rights with other societal concerns and values, such as public health and welfare (Douek, 2021). In algorithmic content moderation, fairness and proportionality are primarily defined by platforms’ policies. But these policies—as well as government regulations—should also be informed by citizens’ preferences for balancing freedom of expression with public health and welfare and other factors. Kozyreva et al. (2023) found that people arrive at relatively consistent valuations of what attributes are relevant for content moderation. Respondents assessed whether and to what degree action should be taken against various instances of harmful misinformation; their willingness to remove posts and even suspend accounts depended on attributes such as the topic of the misinformation (e.g., Holocaust denial, climate-change denial), the severity of harm caused by misinformation, and pattern of past behavior (e.g., first time versus repeated sharing of misinformation). In contrast, attributes of the account itself, such as its partisanship and number of followers, barely influenced respondents’ decisions.

Taken at face value, Kozyreva et al.’s (2023) results suggest that an algorithmic implementation of content-moderation policies should be blinded to personal attributes (e.g., profession, political orientation) and focused on measurable indicators of how much harm the misinformation in question causes as well as whether the account had spread misinformation in the past. Some of the attributes used in algorithmic online content moderation may need to be inferred by machine-learning techniques, thus establishing a hybrid system in which the overarching structure is human designed and top down, and the inference of attribute values is automated and bottom up. As in the case of simple and transparent algorithms discussed above, there should also be a transparent rationale for selecting and omitting attributes. Doing so arguably should facilitate in-depth algorithm audits that can establish reliably causal connections and a better understanding of the algorithms’ design and logic—and complement the study of input–output relationships in black-box approaches (Adler et al., 2018; Feldman et al., 2015; Marx et al., 2019; Yeom et al., 2018).

Blinding algorithms to specific attributes also promises to help protect fundamental rights by curtailing the collection of personal data and the ability to make inferences about users (data and inference minimization). Algorithms, often trained on extensive data, allow for inferences about individuals’ preferences and traits that can then be used for targeted advertising (Lorenz-Spreen et al., 2021). A review of 327 studies found that important personal information, including location, political attitude, and sexual preference, can be reliably inferred from digital fingerprints (Hinds & Joinson, 2018). Furthermore, algorithmic inferences of personality traits from digital fingerprints are more accurate than human judges (Hinds & Joinson, 2019). Some platforms’ algorithms can even create shadow profiles that predict the personal information of individuals who have never used the platform (Garcia, 2017). Should platforms possess this power to predict people’s personalities and sensitive private behaviors? The general public—which may not even be fully aware of just how far algorithmic inferences can reach—clearly opposes the use of personal data and sensitive information for personalization of, for example, political-campaign messages or online news curation (Kozyreva et al., 2021), and a mere 25% of people worldwide trust social media to handle their data responsibly (Newman et al., 2022).

The principle of data minimization (Goldsteen et al., 2022) is an example of blinding algorithms at the data-collection stage. It mandates that personal data collection must be “adequate, relevant and limited to what is necessary in relation to the purposes for which they are processed” (Article 5 of the European Union’s General Data Protection Regulation, or GDPR; European Union, 2016). By minimizing the amount of personal data available for profiling, tracking, or surveillance, it aims to protect individual privacy. We propose extending this principle to inference minimization by specifying which data should be excluded or not collected in the first place on social-media platforms—particularly data that is directly related to sensitive characteristics (e.g., ethnicity, sexual preferences)—and which characteristics should be prohibited from being inferred from other data.

## Role Reversal: Using Algorithms to Blind People

So far, we have explored how human designers can blind algorithms. We now consider the reverse: How can algorithms help humans blind themselves to unwanted information so that they can make fair and accurate judgments? As we have seen, there are numerous examples of people using algorithms in their most general form—a finite set of step-by-step instructions—to blind themselves or others to unwanted information (MacCoun, 2021; Robertson & Kesselheim, 2016; Teichman et al., 2021) in orchestra auditions (Goldin & Rouse, 2000), scientific data analysis, scientific peer review, and job-applicant screening (MacCoun, 2021). We believe that algorithms could be used in many more settings to adaptively blind humans to detrimental information and that this approach presents intriguing but largely unexplored opportunities for research. As an example for a potential research agenda addressing these opportunities, we now apply this perspective to the topic of collective intelligence and examine two scenarios: aggregating the independent judgments of multiple individuals (the “wisdom of crowds”; Herzog et al., 2019; Surowiecki, 2004) and aggregating a single person’s repeated judgments (“wisdom of the inner crowd”; Herzog & Hertwig, 2009, 2014). By doing so we extend our discussion beyond the issues of bias and fairness to also include accuracy in decision-making.

Harnessing the collective wisdom of multiple individuals can be an effective method to improve judgments. This wisdom-of-the-crowd effect has been used in diverse fields, including medical diagnostics (e.g., cancer diagnosis; Kurvers et al., 2016) and economic and political forecasting (Mellers et al., 2014). Tapping into the wisdom of crowds entails eliciting and aggregating accurate, nonredundant judgments from diverse individuals. The role of social influence—in which individuals learn about others’ opinions before or while forming their own—in fostering or thwarting the wisdom-of-the-crowd effect continues to be debated. Nonetheless, studies suggest that deliberately controlling the timing of exposure to social information can boost the wisdom of crowds or collective intelligence more generally. We briefly review two such examples below.

First, forming an independent judgment before considering others’ opinions helps prevent excessive confidence in the accuracy of social information. Koehler and Beauregard (2006) showed that people exposed to an advisor’s numerical estimate before making their own tended to incorporate it into their estimate. This led to an illusion of confirmation, as they did not appear to correct for this influence. Consequently, they expressed greater confidence in the advisor’s estimate than did people who formed their estimate before receiving advice. Second, interaction between people can yield both positive and negative effects on problem-solving and idea generation. When solving problems, people who interact with each other can exploit available answers, resulting in a higher average quality of solutions. However, this social influence also carries a cost: It might limit individual exploration for innovative answers, resulting in lower solution quality compared with independent problem-solving. By only periodically providing individuals with access to others’ ideas and solutions and otherwise shielding them from social information, a balance between these two opposing forces can be struck and overall performance be improved (Bernstein et al., 2018; Paulus et al., 2015).

Blinding people to their own past judgments also promises to enhance individual performance in the wisdom of the inner crowd (Herzog & Hertwig, 2009, 2014). Aggregating nonredundant judgments from the same people can boost accuracy if the task allows for them to be blinded to their own past judgments. This is because the more independent repeated judgments are, the more diverse they are likely to be—an observation consistent with Stroop’s (1932) finding that aggregating either *n* repeated judgments from one person or single judgments from *n* different individuals resulted in equivalent aggregation gains once participants were blinded to their past judgments. Blinding can also be indirectly approximated rather than literally implemented: In a medical-classification task the aggregation of confidence judgments of two repeated classifications was more effective when the same medical image was rotated by 180° for the second viewing (Hasan et al., 2022). It was likely that this intervention reduced the likelihood of participants recognizing the image and recalling their previous classification.

The above examples share a theme: the benefit of blinding people to judgments or ideas from others—or from themselves—at the right moments. Depending on the setting, a good blinding policy can take the form of analog algorithms (i.e., cognitive heuristics) or computer-mediated algorithms. For instance, when seeking a second opinion, a patient can refrain from revealing the first doctor’s diagnosis. This eliminates correlated errors and the illusion of confirmation (see Koehler & Beauregard, 2006). Similarly, a computer system for organizing the double reading of medical X-rays could blind the second rater to the first rater’s diagnosis. Relatedly, medical software could be programmed to automatically rotate images in medical-image-interpretation tasks, thereby reducing error correlation between different experts—or even within the set of a single expert’s judgments (see Hasan et al., 2022). As another example of a computer-mediated algorithm, consider complex problem-solving tasks such as the traveling-salesperson problem studied by Bernstein et al. (2018). This problem involves finding the shortest path among symbols representing cities on a synthetic 2D map presented visually—a computationally very tricky problem. They demonstrated that exposure to others’ solutions once every three rounds led to better results than constant exposure. The ideal mix of exposed and blinded rounds likely depends on the characteristics of the problem and the solver population. With an adequate amount of data, machine-learning techniques could be used to predict effective blinding policies, including honing the decision of which person to expose to which other person’s opinions or solutions (see Burton et al., 2021, for a proof of concept for such algorithms for numerical-estimation tasks). The results and ideas presented here highlight the potential of a general research agenda that asks how one can effectively develop and test adaptive information-blinding architectures that support better human judgment in different domains and tasks.

## Conclusion

At first sight, it seems self-evident that an elegant way to address the harms of implicit and explicit biases in public-policy decisions is to delegate important decisions, such as the allocation of benefits, resources, or opportunities, to seemingly objective algorithms. This approach, however, requires prudence. Rather than exercising dispassionate impartiality, algorithms can perpetuate and amplify existing inequalities and injustices. They do so by replicating biases inherited from their designers or embedded in the data they are trained on. How can the algorithms’ promise of impartiality become reality? Inspired by Rawls’s veil-of-ignorance thought experiment and the concept of deliberate ignorance, we have explored how blinding can help people, institutions, and algorithms to contain the impact of biases on their decisions.

Deliberate ignorance in algorithmic decision-making can be achieved by blinding human-designed algorithms as well as by using algorithms to blind humans. These blinding algorithms can be implemented at different levels—in training data, in model features, and in algorithmic rules. Moreover, algorithms can offer decision-making support by generating recommendations or scores without revealing protected attributes. And by using algorithms for blinding, organizations can identify and address systemic bias, iteratively revising decision-making frameworks to support inclusivity, diversity, and fairness.

The approach we have explored suggests a series of research questions, including these:

- To what extent can top-down human-designed algorithms be leveraged to better blind machines to protected attributes, and how can the success of such blinding methods be audited?
- How can algorithmic blinding, including specifying what ought to be hidden, be informed by psychological studies across different domains (e.g., content moderation, hiring, and political advertising)? Do people have consistent and converging preferences about attributes and information that should be hidden?
- How can the success of machine blinding in various operating environments be empirically demonstrated rather than merely assumed?
- What regulations for machine-based decision processes and blinding principles will be effective, efficient, and normatively appropriate? How can regulations ensure a balance between different goals and concerns, such as fostering programmers’ creativity in designing blinding methods, avoiding paternalism when making decisions about blinding, and attenuating implicit and explicit biases?
- How might organizations employ the strategy of blinding their members to mitigate organizational problems like diffusion of responsibility and suppression of disagreement? To what extent can the increasingly digital workplace serve as a foundation for the implementation of blinding algorithms?
- Are the reasons given by Greenwald et al. (2022) sufficient to explain blinding’s infrequent use, and, if so, how can they be overcome? Greenwald et al. (2022) suggested three possible reasons that blinding is not used more often: the assumption that people can ignore potentially biasing information; the fear that blinding diminishes people’s ability to rely on their experience; and organizational inertia.

We hope that these and related research questions will encourage new ideas, research, and cross-talk among computer scientists, psychologists, legal scholars, and other experts. Much is at stake. It does not require clairvoyance to foresee that algorithmic decision-making will become even more prevalent. Concurrently, another defining trend of our time is that countries with more inequality, among other properties, are more vulnerable to populism (Pástor & Veronesi, 2021). In light of this, an important question arises: to what extent can we design fair and effective algorithmic decision-making systems—and human systems, for that matter—that can work as equalizing forces? And how can the veil of ignorance, research on deliberate ignorance, and blinding methods contribute to this goal?

## Transparency

*Action Editor:* Mirta Galesic

*Editor:* Interim Editorial Panel

*Author Contributions*

The first and second authors contributed equally to this work.

**Ralph Hertwig:** Conceptualization; Writing – original draft preparation; Writing – review & editing.

**Stefan M. Herzog:** Conceptualization; Writing – original draft preparation; Writing – review & editing.

**Anastasia Kozyreva:** Conceptualization; Writing – original draft preparation; Writing – review & editing.
